# Phylogenetic and Transcription Analysis of Chrysanthemum WRKY Transcription Factors

**DOI:** 10.3390/ijms150814442

**Published:** 2014-08-19

**Authors:** Aiping Song, Peiling Li, Jiafu Jiang, Sumei Chen, Huiyun Li, Jun Zeng, Yafeng Shao, Lu Zhu, Zhaohe Zhang, Fadi Chen

**Affiliations:** 1 College of Horticulture, Nanjing Agricultural University, Nanjing 210095, China; E-Mails: aiping_song@aliyun.com (A.S.); 2011204026@njau.edu.cn (P.L.); jiangjiafu@njau.edu.cn (J.J.); chensm@njau.edu.cn (S.C.); 2010204024@njau.edu.cn (H.L.); 2010104110@njau.edu.cn (J.Z.); 2010104109@njau.edu.cn (Y.S.); 2011104109@njau.edu.cn (L.Z.); 2012104099@njau.edu.cn (Z.Z.); 2 Jiangsu Province Engineering Lab for Modern Facility Agriculture Technology & Equipment, Nanjing 210095, China

**Keywords:** *Chrysanthemum morifolium*, phylogenetic analysis, stress response, transcription pattern

## Abstract

WRKY transcription factors are known to function in a number of plant processes. Here we have characterized 15 *WRKY* family genes of the important ornamental species chrysanthemum (*Chrysanthemum morifolium*). A total of 15 distinct sequences were isolated; initially internal fragments were amplified based on transcriptomic sequence, and then the full length cDNAs were obtained using RACE (rapid amplification of cDNA ends) PCR. The transcription of these 15 genes in response to a variety of phytohormone treatments and both biotic and abiotic stresses was characterized. Some of the genes behaved as would be predicted based on their homology with *Arabidopsis thaliana WRKY* genes, but others showed divergent behavior.

## 1. Introduction

Chrysanthemum (*Chrysanthemum morifolium* Ramat.) is a leading ornamental species, second only to the rose in terms of its market value [1]. In 2010, more than two billion cut chrysanthemum stems were produced in China, and almost the same number in Japan [2]. Since the major constraints faced by chrysanthemum producers are a range of biotic and abiotic stresses, enhancing the crop’s resistance/tolerance to these is an important breeding aim.

The WRKY family is prominent among plant transcriptional regulators, so named because of the presence of the characteristic peptide sequence WRKYGQK [3]. Sweet potato SPF1 was the first WRKY protein to have been isolated [4]. Based on the number of WRKY domains present and the structure of the protein’s zinc finger motifs, three major groups of WRKY proteins have been recognized [5]. Group I proteins contain two WRKY domains and a C_2_H_2_ zinc finger motif, group IIs a single WRKY domain and a C_2_H_2_ zinc finger motif, and group IIIs a C_2_HC zinc finger motif [5]. WRKY transcription factors are known to be involved in the regulation of a number of aspects of plant growth and development, as well as in the response to stress [6,7,8,9]. In *Arabidopsis thaliana*, for example, they participate in the response to low temperature, drought and salinity [10], while others have been implicated in signaling in the context of pathogen infection [11,12,13,14,15] and herbivore attack [16]. They have been shown to interact with phytohormones, especially salicylic acid (SA) and jasmonate (JA) [17,18].

As yet, however, the various activities of WRKY proteins in chrysanthemum have not been explored. Here, we report the isolation of 15 chrysanthemum WRKY transcription factors, based on a set of transcriptomic data, and have analyzed the effect of various stress and phytohormone treatments on their level of transcription.

## 2. Results and Discussion

### 2.1. The WRKY Gene Content of Chrysanthemum

**Figure 1:**
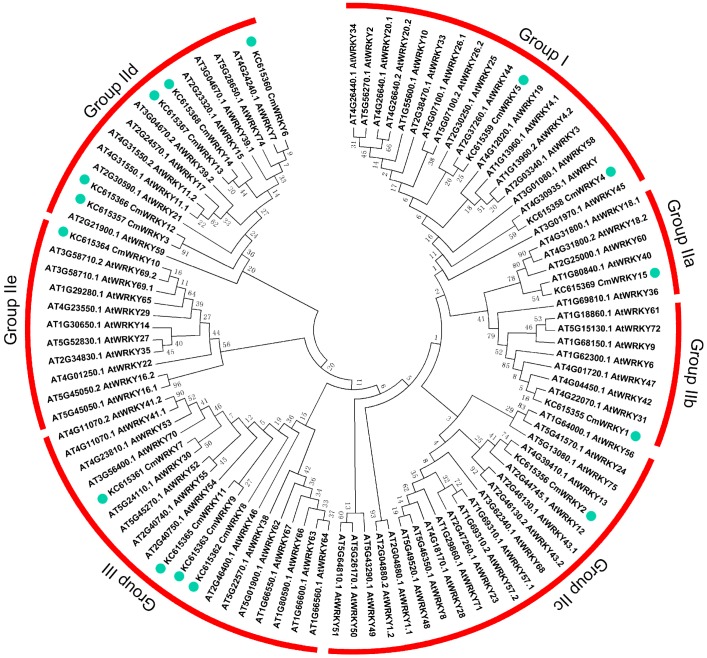
An unrooted phylogenetic tree of the WRKY peptide sequences of chrysanthemumand *A. thaliana*. Sequences were aligned using ClustalW and the phylogeny constructed using the neighbor-joining method. The red arcs indicate the various groups (and subgroups) defined by the presence/absence of known WRKY domains. Dots indicate likely homologs.

**Figure 2:**
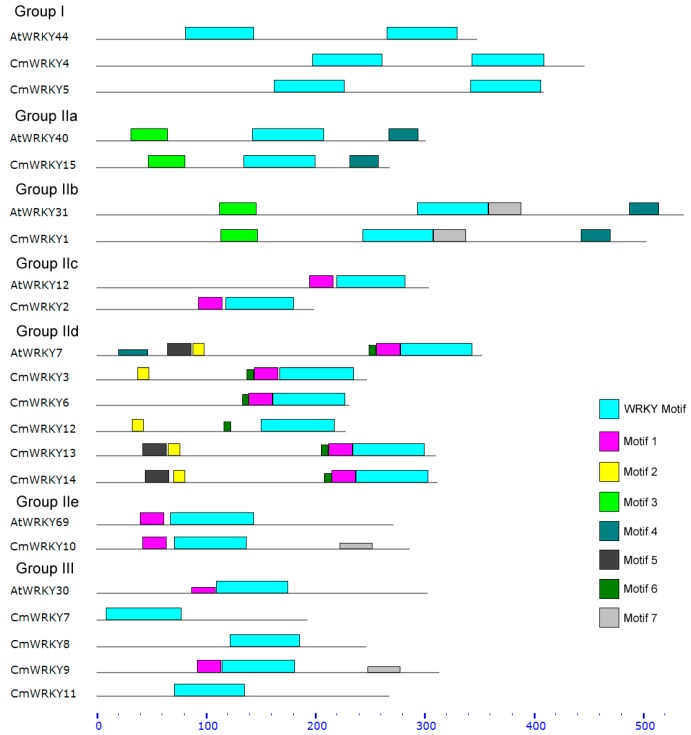
The amino acid motifs present in the CmWRKY and AtWRKY proteins, as determined by Meme 4.8.1 software [19]. The cyan boxes represent WRKY motif, and other colored boxes each represent a specific motif with uncharacterized function.

The 15 *WRKY* sequences isolated were designated *CmWRKY1* through *CmWRKY15* (GenBank: KC615355–KC615369). The full length cDNAs varied in length from 757 to 1750 bp, and their predicted protein products comprised between 193 and 504 residues. Full details of the *CmWRKY* sequences are given in Table 1. A combination of sequence comparison, phylogenetic and structural analyses suggested that the 15 *CmWRKY* genes were distributed across the three known *WRKY* groups (Figure 1), and a schematic overview of the core motifs present is shown in Figure S1. There was a high degree of homology between the motifs present in the CmWRKYs and those in the AtWRKYs (Figure 2). Motif 6 only featured in Group IId, while motif 3 was present in both Groups IIa and IIb (Figure S1).

**Table 1:** *CmWRKY* gene sequences and the identity of likely *A. thaliana* homologs*.*

Gene	GenBank Accession No.	cDNA Length (bp)	Amino Acids Length (aa)	AtWRKY Orthologs	Locus Name	*E*-Value
*CmWRKY1*	KC615355	1750	504	*AtWRKY6*	AT1G62300	5e-86
*CmWRKY2*	KC615356	823	200	*AtWRKY13*	AT4G39410	2e-47
*CmWRKY3*	KC615357	928	248	*AtWRKY11*	AT4G31550	3e-38
*CmWRKY4*	KC615358	1608	447	*AtWRKY32*	AT4G30935	3e-68
*CmWRKY5*	KC615359	1668	410	*AtWRKY44*	AT2G37260	5e-74
*CmWRKY6*	KC615360	1119	232	*AtWRKY21*	AT2G30590	2e-56
*CmWRKY7*	KC615361	757	193	*AtWRKY41*	AT4G11070	2e-31
*CmWRKY8*	KC615362	1019	247	*AtWRKY41*	AT4G11070	5e-30
*CmWRKY9*	KC615363	1331	314	*AtWRKY46*	AT2G46400	8e-37
*CmWRKY10*	KC615364	1216	287	*AtWRKY65*	AT1G29280	3e-49
*CmWRKY11*	KC615365	1117	268	*AtWRKY70*	AT3G56400	6e-31
*CmWRKY12*	KC615366	875	229	*AtWRKY17*	AT2G24570	3e-24
*CmWRKY13*	KC615367	936	311	*AtWRKY7*	AT4G24240	6e-65
*CmWRKY14*	KC615368	942	313	*AtWRKY7*	AT4G24240	1e-53
*CmWRKY15*	KC615369	941	268	*AtWRKY40*	AT1G80840	1e-43

Orthology detection is critically important for accurate functional annotation, and has been widely used to facilitate studies on comparative and evolutionary genomics. A trade-off between sensitivity and specificity in orthology detection was observed, with BLAST (Basic Local Alignment Search Tool)-based methods characterized by high sensitivity, and phylogeny-based methods by high specificity [20], so the relationship between the chrysanthemum and *A. thaliana* *WRKY* genes was analyzed using both BLAST (which delivers a local sequence alignment) and by a rooted phylogenetic tree (global sequence alignment) (Table 1, Figure 1). Some inconsistencies were noted: for example, *CmWRKY7* and *AtWRKY69* appeared to be closely related according to the phylogenetic analysis, but the BLAST comparison predicted that the closest *A. thaliana* sequence to *CmWRKY7* was *AtWRKY41*. Gene function analysis may therefore be needed to conclude which of these relationships is the more likely to be valid.

### 2.2. Transcription Profiling of CmWRKY Genes

**Figure 3:**
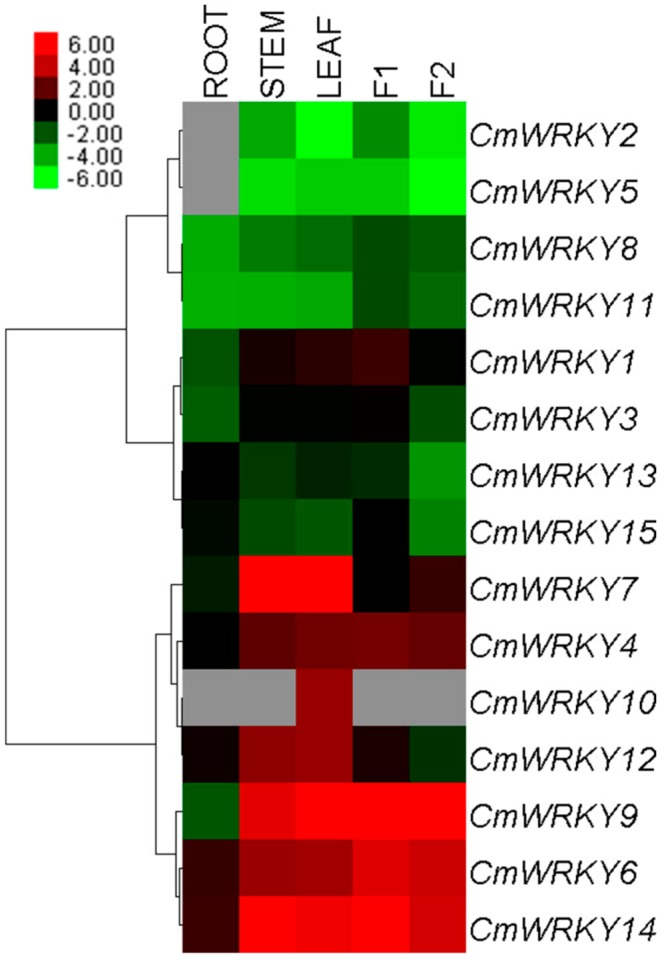
Differential transcription of *CmWRKY* genes. F1: tube florets, F2: ray florets at budding stage. Green indicates lower and red higher transcript abundance compared to the relevant control. Grey blocks indicate that transcription was not detected.

The 15 *CmWRKY* genes were differentially transcribed throughout the plant (Figure 3). The transcript abundance of *CmWRKY14* was more than three orders of magnitude higher than that of *CmWRKY2* in the leaf, while *CmWRKY10* transcript was only detectable in the leaf under non-stressed conditions. Neither *CmWRKY2* nor *CmWRKY5* transcript was present in the root. The level of transcription shown by *CmWRKY* in tube florets was at least double that present in ray florets at budding stage, besides *CmWRKY7* was exception.

### 2.3. The Transcription of CmWRKY Genes in Plants Challenged by Phytohormones and Abiotic Stress

**Figure 4:**
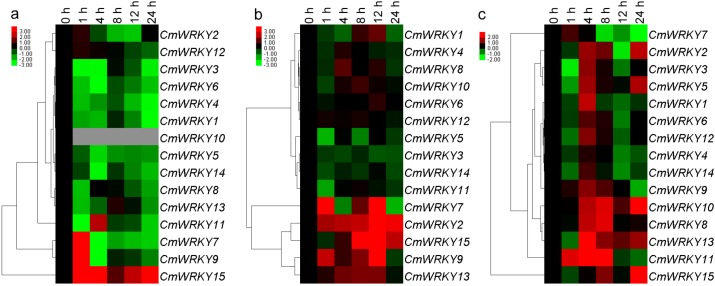
Differential transcription of *CmWRKY* genes in leaves as induced by the exogenous supply of (**a**) abscisic acid (ABA); (**b**) methyl jasmonate (MeJA) and (**c**) salicylic acid (SA). Green indicates lower and red higher transcript abundance compared to the relevant control. Grey blocks indicate that transcription was not detected.

Twelve of the 15 genes were down-regulated by exogenous ABA (abscisic acid) (the exceptions were *CmWRKY7*, *9* and *15*), while the abundance of *CmWRKY10* transcript was below the level of detection. *CmWRKY15* was induced by this treatment, while *CmWRKY7* and *9* were also induced, but only after exposure of least 1 h (Figure 4a). None of *CmWRKY1*, *4*, *6*, *8*, *10* or *12* were responsive to MeJA (methyl jasmonate) treatment, but *CmWRKY2*, *9*, *13* and *15* were induced, while *CmWRKY3*, *5*, *11* and *14* were all repressed (Figure 4b). Eleven of the genes (the exceptions were *CmWRKY7*, *9*, *10* and *11*) were repressed after 1 h exposure to SA treatment, but their transcription was triggered after 4 h (Figure 4c).

**Figure 5:**
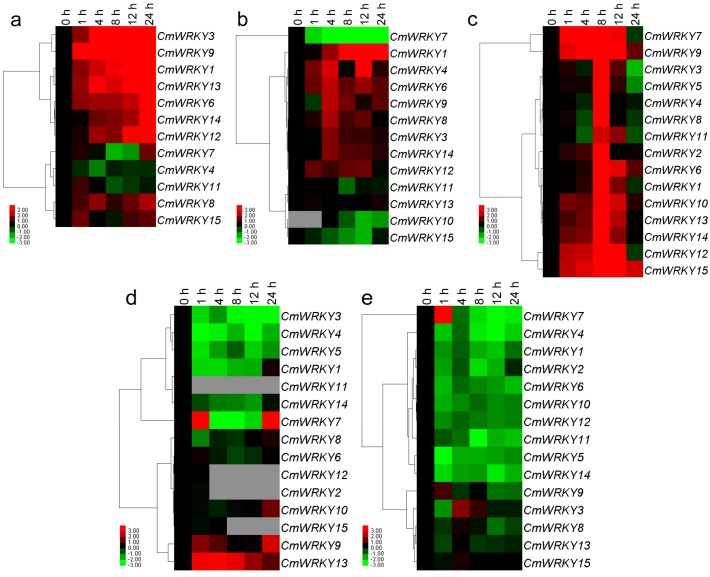
Differential transcription of *CmWRKY* genes as induced by abiotic treatments at the seedling stage. (**a**) roots in salinity; (**b**) roots in moisture stress; (**c**) leaves in low temperature; (**d**) leaves in high temperature and (**e**) leaves undergo wounding. Green indicates lower and red higher transcript abundance compared to the relevant control. Grey blocks indicate that transcription was not detected.

*CmWRKY1*, *3*, *6*, *9*, *12*, *13* and *14* were all up-regulated in the root by salinity stress, while *CmWRKY4* was down-regulated. The transcript abundance of *CmWRKY7*, *11* and *15* was enhanced after 1 h of exposure, but later fell back (Figure 5a). The effect of moisture stress was to up-regulate *CmWRKY1*, *3*, *4*, *6*, *8*, *9*, *12* and *14* in the root by various amounts, while *CmWRKY7* transcription was markedly suppressed. Transcription of *CmWRKY10* was noted after 4 h of exposure to PEG (polyethylene glycol), but was not transcribed in non-stressed roots (Figure 5b). All of the *CmWRKY* genes were induced by exposure to low temperature, with the peak transcript abundance occurring after 8 h (Figure 5c). With the exception of *CmWRKY7*, *9* and *13*, the genes were all down-regulated by high temperature; the abundance of *CmWRKY2*, *11*, *12* and *15* transcript was below the level of detection (Figure 5d). Apart from *CmWRKY7* and *9*, the genes were all down-regulated by wounding (Figure 5e).

### 2.4. Differential Responses of the CmWRKY Genes to Biotic Stress

**Figure 6:**
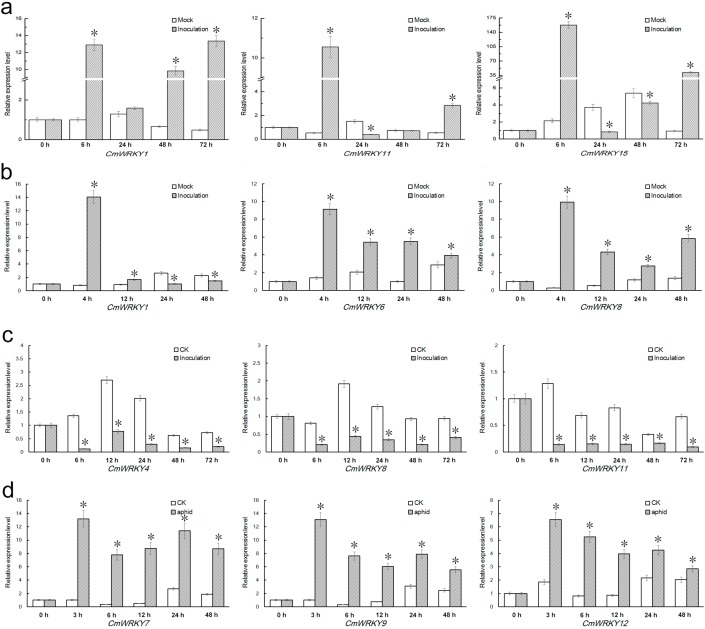
Differential expression patterns of the *CmWRKY* genes in leaves to biotic stress. (**a**) inoculation with *A. tenuissima*; (**b**) inoculation with *F. oxysporum*; (**c**) inoculation with *P. horiana*; and (**d**) infestation with the aphid *M. sanbourni*. Asterisks indicate significant differences (*p* < 0.05) between treatment and control plants.

*CmWRKY1*, *11* and *15* were strongly induced by the presence of *A. tenuissima* inoculum, particular *CmWRKY15*, for which the level of transcript was some 80 fold higher than that of the non-inoculated control after 6 h (Figure 6a). *CmWRKY1*, *6* and *8* were all induced by about 10 fold when assayed 4 h after inoculation with *F. oxysporum* (Figure 6b). *CmWRKY4*, *8* and *11* were markedly suppressed by *P. horiana* inoculation (Figure 6c), while *CmWRKY7*, *9* and *12* responded positively to aphid infestation (Figure 6d). The expression changes of other *CmWRKY* genes were less than two fold in magnitude.

### 2.5. Comparison of the Expression Pattern between CmWRKY Genes and Correlated Arabidopsis Homologs

Accumulating evidence suggests that many *WRKY* genes are involved in the regulation of plant development and their stress response [3,21]. Here, it was clear that the *CmWRKY* genes differed from one another with respect to their tissue specificity and inducibility. The various stress treatments affected the transcription level of different combinations of the 15 *CmWRKY* genes, implying that most of them do contribute to the stress responses of chrysanthemum. The *A. thaliana* genes *AtWRKY6* [22], *40* [23], *41* [14] and *70* [24] all participate in the defense against pathogen attack, and their homologs (as determined by BLAST) *CmWRKY1*, *8*, *11* and *15* were similarly induced by pathogen inoculation. On the other hand, *AtWRKY31* (the homolog of which, based on phylogeny, is *CmWRKY1*) has no known involvement in the disease response. *AtWRKY17* is up-regulated by salinity stress [25], as is its homolog *CmWRKY12*. *AtWRKY11*, *17* and *70*, all of which are induced by wounding, drought and low temperature [26,27,28], also play a role in the interaction between SA and JA [29,30]. Their orthologs in chrysanthemum (respectively, *CmWRKY3*, *12* and *11*) were also induced by moisture stress (PEG treatment) and low temperature, but were down-regulated by wounding; and in converse response to exogenous SA and JA. *AtWRKY70* is activated by SA but suppressed by JA [29]; its constitutive expression improves the level of resistance to biotrophic, but not to necrotrophic fungi [17,31]. Its chrysanthemum homolog *CmWRKY11* was inducible by inoculation with necrotrophic fungi and the exogenous supply of SA, but suppressed by biotrophic fungi and JA, which is rather different from the behavior of *AtWRKY70*. *AtWRKY40* is an important component of WRKY-mediated ABA signaling, and exogenous ABA treatment reduces both its transcription and translation [32]. *CmWRKY15*, its chrysanthemum homolog, was notably up-regulated by exogenous ABA, which again suggests that some pairs of *WRKY* homologs have evolved different functionality between *A. thaliana* and chrysanthemum. The function of *AtWRKY13*, *21* and *32* is not well understood, so little can be concluded by comparing these with their chrysanthemum homologs, respectively, *CmWRKY3*, *12* and *11.*

## 3. Experimental Section

### 3.1. Plant Materials

Cuttings of the cut flower chrysanthemum cultivar “Jinba”, maintained by the Chrysanthemum Germplasm Resource Preserving Center (Nanjing Agricultural University, Nanjing, China), were rooted in vermiculite in the absence of fertilizer in a greenhouse. After 14 days, they were transplanted into growth substrate, in preparation for exposure to a range of stress and phytohormone treatments.

### 3.2. Isolation and Sequencing of Full-Length CmWRKY cDNAs

Total RNA was isolated from leaves, stems, roots or florets using the RNAiso reagent (TaKaRa, Tokyo, Japan), following the manufacturer’s instructions. The first cDNA strand was synthesized from 1 μg total RNA using M-MLV (Moloney murine leukemia virus) reverse transcriptase (TaKaRa), according to the manufacturer’s instructions. Primer pairs (listed in Table S1) were designed to amplify internal *CmWRKY* fragments, based on sequences identified in a chrysanthemum transcriptome database (unpublished data). The sequences of the resulting amplicons were used to derive full length cDNAs via 5'- and 3'-RACE PCR. For the 3' reaction, the first cDNA strand was synthesized using the dT adaptor primer dT-AP, and this was followed by a nested PCR based on the primer pair CmWRKYx-3-F1/F2 and the adaptor primer AP (Table S2). For the 5' reaction, the primers consisted of AAP and AUAP (provided with the 5' RACE System kit v2.0, Invitrogen, Carlsbad, CA, USA), along with the gene-specific primer pairs CmWRKYx-5-F1/F2 (Table S3). Where required, amplicons were purified using an AxyPrep DNA Gel Extraction kit (Axygen, Hangzhou, China) and cloned into pMD19-T (TaKaRa) for sequencing. Finally, 15 pairs of gene-specific primers (CmWRKYx-ORF-F/R, Table S4) were elaborated to amplify the full open reading frame sequences.

### 3.3. Phylogeny of WRKY Sequences

*A. thaliana* WRKY sequences were downloaded from the Database of Arabidopsis Transcription Factors (DATF) [33], and combined with the newly acquired *CmWRKY* sequences to perform a multiple alignment analysis based on ClustalW software [34]. The subsequent phylogenetic analysis utilized the Neighbor-Joining method, and a graphical representation was produced with the help of MEGA5 software [35]. Internal branching support was estimated using 1000 bootstrap replicates. The MEME v4.8.1 program [19] served to identify the motifs present in the 15 CmWRKY proteins, using the parameter settings suggested by Huang [21], and retaining only motifs associated with an *E* value <e^−5^.

### 3.4. Plant Treatments

The tissue-specific and treatment-induced transcription profiles of the 15 *CmWRKY* genes was explored in young seedling roots, stems and leaves, and in the tube and ray florets of inflorescences at the bud stage. A variety of abiotic stresses was imposed, namely salinity (200 mM NaCl), moisture deficit (20% *w*/*v* polyethylene glycol (PEG6000)) [36], low temperature (4 °C), high temperature (40 °C) and wounding. Plants were also inoculated separately with three different fungi, namely the necrotroph *Alternaria tenuissima*, the biotroph *Puccinia horiana* and the hemibiotroph *Fusarium oxysporum*; further plants were infested with the aphid *Macrosiphoniella sanbourni*.

For the NaCl and PEG6000 assays, young plants were transferred to a liquid medium containing the stress agent, and the roots were sampled at various time points. Other seedlings were exposed to a period of either 4 or 40 °C in a chamber delivering a 16 h photoperiod and 50 μmol·m^−2^·s^−1^ light, after which the second true leaves were sampled [37]. The wounding treatment involved cutting the second true leaf. The phytohormone treatments involved spraying the leaves with either 50 μM abscisic acid (ABA) [38], 1 mM methyl JA (MeJA) [39] or 200 μM SA [40]. Plants were sampled prior to the stress treatment and then after one, four, eight, 12 and 24 h.

Spore suspensions of *A. tenuissima* and *F. oxysporum* were obtained from a 14 day old PDA (potato dextrose agar) cultures, to which 10 mL of sterile water had been added; the spore suspension was then filtered through four layers of cheesecloth, and the spore concentration adjusted to 10^7^ per mL using a haemocytometer [41,42]. Leaves were sampled before inoculation with *A. tenuissima* and then after six, 24, 48 and 72 h; the roots were sampled prior to *F. oxysporum* inoculation, and then after four, 12, 24 and 48 h. Infection with *P. horiana* was achieved using the spray method described by Zandvoort *et al.* [43] with the following modifications: Briefly, the infected leaves containing white rust pustules were cut into small pieces and were dispersed in deionized water and filtered through medical gauze to remove any plant debris. The concentration of the pathogenic spore suspension was then adjusted using a hemacytometer slide to a concentration of 10^6^ zoosporangia·mL^−1^ with deionized water containing one drop of Tween 20 before application to the plants until run-off, using a hand-held sprayer. Leaves were sampled before inoculation and then after six, 12, 24, 48 and 72 h. The method used for aphid infestation followed [44] with the following minor modifications: Briefly, twenty aphids in two instar nymphs were transferred to the plants by a soft brush. A 25 cm long × 12 cm diameter polyester cylinder capped with fine gauze was placed over each plant to prevent any movement of aphids between adjacent plants. Leaves were sampled prior to infestation, and then after three, six, 12, 24 and 48 h.

After sampling, all the material was snap frozen in liquid nitrogen and stored at −70 °C. Each treatment was replicated three times.

### 3.5. Real-Time Quantitative PCR (qPCR)

qPCRs were performed on Mastercycler ep realplex device (Eppendorf, Hamburg, Germany). Each 20 μL qPCR contained 10 μL SYBR^®^ Premix Ex Taq™ II (Takara), 0.4 μL of each primer (10 μM), 4.2 μL H_2_O and 5 μL cDNA template. The PCR cycling regime comprised an initial denaturation (95 °C/2 min), followed by 40 cycles of 95 °C/10 s, 55 °C/15 s, 72 °C/20 s. A melting curve analysis was conducted following each assay to confirm the specificity of the amplicons. Gene special primers (sequences shown in Table S5) were designed using PRIMER3 RELEASE 2.3.4 [45], and the EF1α gene was used as a reference sequence. Relative transcript abundances were calculated by the 2^−ΔΔ*C*t^ method [46].

### 3.6. Data Analysis

The relative transcription levels of each *CmWRKY* were log_2_ transformed, and the profiles compared using Cluster v3.0 software [47] and visualized using Treeview [48]. SPSS v17.0 software (SPSS Inc., Chicago, IL, USA) was used for all statistical analyses.

## 4. Conclusions

The comparative analysis of functionality in chrysanthemum and *A. thaliana* suggests that the local alignment method is superior to phylogenetic analysis in predicting functional homology. The present study has documented the transcription behavior of 15 *CmWRKY* genes in response to a range of stress treatments. These data provide a basis for identifying which individual *CmWRKY* genes might be usefully targeted for improving the stress tolerance of chrysanthemum.

## Supplementary Files

Supplementary File 1
