# Supplementary figures and images for: Phylogenetic and Transcription Analysis of Chrysanthemum WRKY Transcription Factors
Source: Int J Mol Sci. 2014 Aug 19;15(8):14442–55. doi: 10.3390/ijms150814442 (PMC4159861; doi:10.3390/ijms150814442)

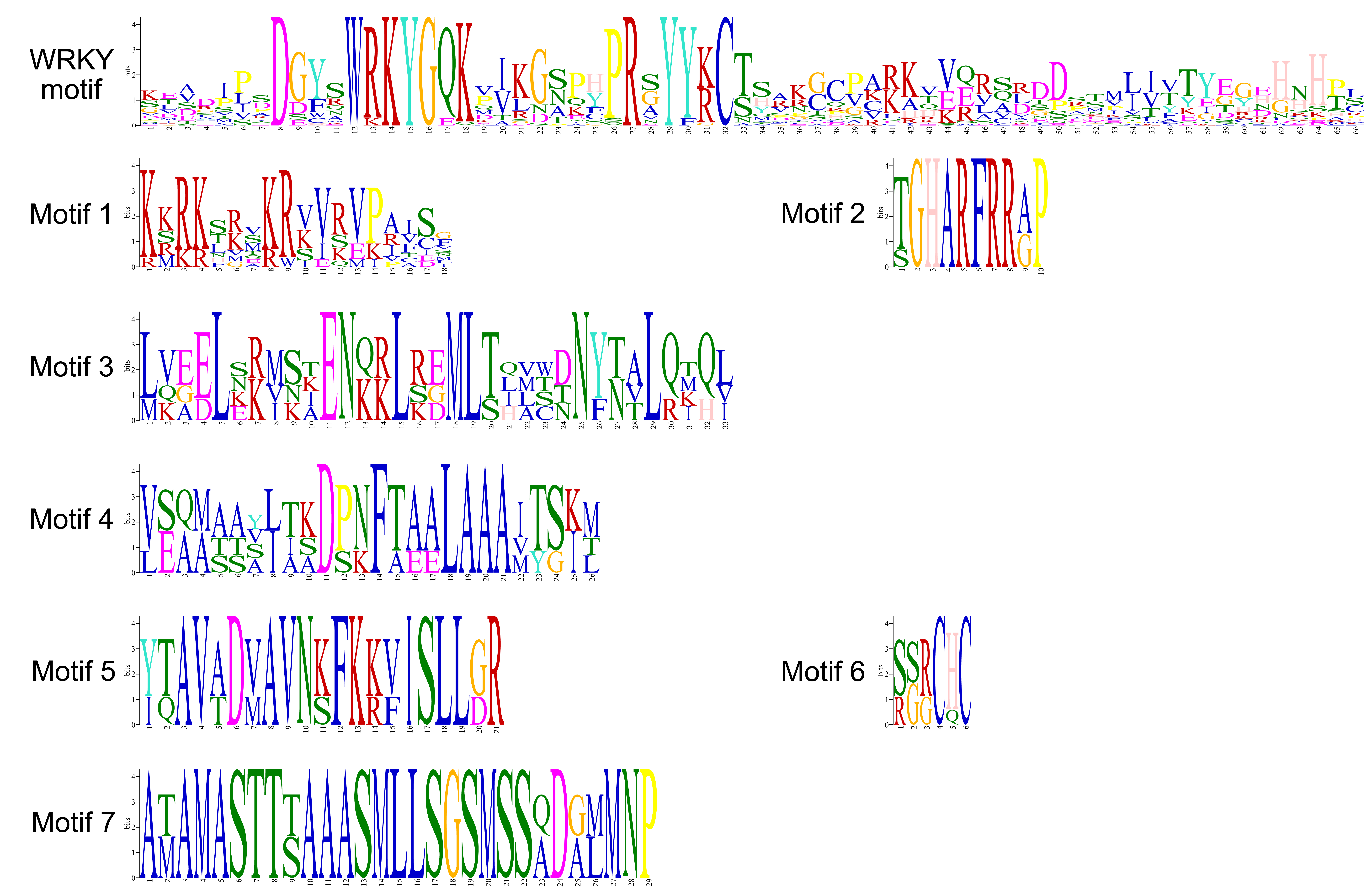

Supplement: Supplementary File 1 [file ijms-15-14442-s001.zip › Supplementary Fig. S1.tif]
